# Durability of Humoral Responses after an Adapted SARS-CoV-2 mRNA Vaccine Dose in Hemodialysis Patients

**DOI:** 10.3390/vaccines12070738

**Published:** 2024-07-03

**Authors:** Louise Benning, Marie Bartenschlager, Heeyoung Kim, Christian Morath, Martin Zeier, Paul Schnitzler, Ralf Bartenschlager, Claudius Speer

**Affiliations:** 1 Department of Nephrology, Heidelberg University, 69120 Heidelberg, Germanychristian.morath@med.uni-heidelberg.de (C.M.); martin.zeier@med.uni-heidelberg.de (M.Z.); 2 Medical Faculty Heidelberg, Department of Infectious Diseases, Molecular Virology, Heidelberg University, 69120 Heidelberg, Germany; marie.bartenschlager@med.uni-heidelberg.de (M.B.); heeyoung.kim@med.uni-heidelberg.de (H.K.); ralf.bartenschlager@med.uni-heidelberg.de (R.B.); 3 Medical Faculty Heidelberg, Department of Infectious Diseases, Virology, Heidelberg University, 69120 Heidelberg, Germany; paul.schnitzler@med.uni-heidelberg.de; 4 German Center for Infection Research (DZIF), 69120 Heidelberg, Germany; 5 Division Virus-Associated Carcinogenesis, German Cancer Research Center (DKFZ), 69120 Heidelberg, Germany; 6 Medical Faculty Heidelberg, Department of Internal Medicine III (Cardiology, Angiology, and Pneumology), Heidelberg University, 69120 Heidelberg, Germany

**Keywords:** COVID-19, hemodialysis, humoral response, BNT162b2, bivalent vaccination, SARS-CoV-2

## Abstract

We recently showed that an adapted SARS-CoV-2 vaccine with wildtype and BA.4/BA.5 Omicron subtype epitopes induced a broad short-term immune response in hemodialysis patients. Antibodies with protective capacity were boosted significantly after a follow-up period of 3 weeks following a fifth vaccine dose. However, data on the longevity of the humoral response after bivalent vaccination are lacking but urgently needed to make recommendations for further booster vaccinations in this patient group. This study is an extension of our previously published data including 40 patients on hemodialysis with a follow-up period of 12 months after an adapted booster vaccine dose. We performed a detailed characterization of humoral immune responses and assessed breakthrough infections. In addition, the severity of breakthrough infections was assessed using an established grading system. Anti-S1 IgG and surrogate neutralizing antibodies significantly decreased during the period of 12 months (*p* < 0.01 and *p* < 0.001, respectively). Live-virus neutralizing antibodies against the wildtype and the BA.5 subtype also significantly decreased over time (*p* < 0.01 and *p* < 0.01, respectively). However, even 12 months after administration of the adapted vaccine dose, all 40/40 (100%) of hemodialysis patients showed detectable SARS-CoV-2 wildtype neutralization activity, with 35/40 (88%) also exhibiting detectable BA.5 subtype neutralization activity. During follow-up, 13/40 (33%) patients contracted a SARS-CoV-2 breakthrough infection, among which 12 cases were categorized as asymptomatic or mild, while only 1 case was classified as moderate disease activity. Thus, bivalent booster vaccination seems to induce a sustained immune response in hemodialysis patients over a period of 12 months with breakthrough infections occurring frequently but predominantly manifesting as asymptomatic or mild.

## 1. Introduction

Severe cases of COVID-19 disease have been shown to be significantly more common among immunocompromised individuals, such as hemodialysis patients, as compared to the general population, particularly before the introduction of SARS-CoV-2 vaccines [1]. For this reason, one of the biggest challenges at the beginning of the pandemic and at the start of vaccination was to protect these vulnerable cohorts as effectively as possible from severe courses of the disease. However, as immunocompromised patient cohorts were not included in large multicenter studies at the beginning of the pandemic, it was unclear for a long time to what extent these patients could be effectively protected by the available vaccines. However, it soon became apparent that a humoral and cellular immune response could also be established in hemodialysis patients after initial complete standard immunization and especially after successful booster vaccination [2]. Nonetheless, the mortality and morbidity of dialysis patients remained high even after vaccination, especially at times when the Alpha and Delta variants predominated [3,4]. The reasons for the limited immune response of patients on hemodialysis are not fully understood. A probable cause is an accelerated aging of the immune system caused by chronic inflammation due to the persistence of uremic toxins, which consecutively leads to impaired cellular immunity. For example, we were able to show that the differentiation of different T cell subpopulations such as responder T cells and regulatory T cells appears to be significantly impaired in dialysis patients, which in turn could also have an influence on the vaccination response of these patients [5]. 

With the attenuation of the original virus strain and the global vaccination campaign that began in late 2020, COVID-19-related mortality has gradually decreased over time. However, patients with declining kidney function and those on hemodialysis have shown an impaired response to various SARS-CoV-2 vaccines and recommended vaccination strategies compared to healthy individuals [6]. In particular, neutralizing antibody levels that are highly predictive of protection against severe COVID-19 disease courses were shown to be significantly lower in hemodialysis patients and kidney transplant recipients [7,8]. Neutralizing antibodies against SARS-CoV-2 are specific immunoglobulins that play a central role in antiviral defense. They bind with high affinity to the viral spike protein, in particular to the receptor binding domain, thereby blocking interaction with the ACE2 receptor on host cells. This binding inhibition prevents viral fusion and entry into the cell, effectively inhibiting viral replication [9]. With regard to the response of various vaccination programs, most large studies therefore focused on the detection of these antibodies against various variants.

Especially in hemodialysis patients, the emergence of variants of concern with humoral and cellular immune escape raised concerns about potential breakthrough infections and more severe disease courses. Although near-complete protection against severe courses of COVID-19 has been demonstrated in the first few months after administration of a vaccine dose for the majority of hemodialysis patients, long-term protection against both, breakthrough infections and severe COVID-19 disease courses, has not yet been fully clarified. Measures to combat declining protection against COVID-19 breakthrough infections in immunocompromised individuals include variant-adapted booster vaccinations comprising spikes from the SARS-CoV-2 variants [10]. Bivalent SARS-CoV-2 vaccines contain a combination of spike epitopes of both the wildtype and the BA.4/BA.5 subtype. These adapted vaccines have been shown to induce a broad vaccine response in both healthy individuals and hemodialysis patients [11,12,13,14]. Especially in the healthy population, it could be clearly shown that neutralizing antibodies against different Omicron subvariants could be significantly increased by the bivalent mRNA COVID vaccination compared to non-adapted vaccines. This also led to a significant reduction in both the rate of breakthrough infections and the rate of hospitalizations. However, as known from previous vaccination studies, the vaccination response was also significantly reduced in these healthy individuals after just a few months.

We have recently shown that in patients without prior SARS-CoV-2 infection, a fifth booster dose of the bivalent mRNA vaccine significantly increases antibody levels against different spike epitopes of the variants it contains [15]. Although the recipient’s SARS-CoV-2 specific immune response was enhanced significantly, long-term data on breakthrough infections and disease severity after a bivalent vaccination are still lacking. These data on the durability of humoral responses after an adapted SARS-CoV-2 vaccination in patients on hemodialysis, however, are crucial for adapting and developing further booster vaccination strategies. To address this issue, we performed a 12-month follow-up of hemodialysis patients who had been vaccinated with a fifth booster dose of the bivalent mRNA vaccine to investigate the longevity of humoral immune responses over time. This study is an extension of our previously published data on short-term immune responses before and three weeks after an additional adapted vaccine dose. Now, we performed a longitudinal assessment of variant-specific protective antibodies and monitored breakthrough infections and their severity during the follow-up period of 12 months.

## 2. Materials and Methods

### 2.1. Study Design

**Figure 1:**
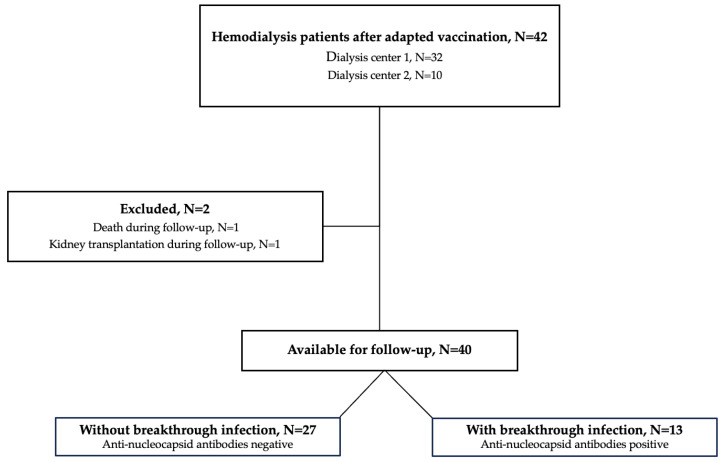
Flow chart of the study population. N, number.

In this study, we performed a 12-month follow-up of our previously published data on humoral immune responses before and 3 weeks after an adapted fifth vaccine dose including wildtype, BA.4 and BA.5 epitopes (Pfizer) [15]. The study cohort of 42 hemodialysis patients, recruited in Heidelberg, Germany, has already been described in detail elsewhere [15]. Forty patients were eligible for study participation and two patients were lost during follow-up (Figure 1). Of these two patients, one patient was excluded because he died during the follow-up and one patient was excluded because he received a kidney transplant during the follow-up. We measured antibodies against the wildtype spike protein as well as different neutralizing antibodies against SARS-CoV-2 variants. Prior breakthrough infections during the last year were either confirmed or excluded by serum antibodies against the nucleocapsid protein [16,17,18]. 

In addition, COVID-19 disease severity of breakthrough infections was assessed during follow-up using an established grading system [19,20]. After the 12-month follow-up, 13 patients were categorized in the group with breakthrough infection and 27 patients in the group without breakthrough infection. Asymptomatic/mild infections were classified as no or only mild symptoms such as fever, cough, change in sense of smell and taste, no occurrence of dyspnea and no need for hospitalization. Moderate disease activity was classified as clinical or radiographic evidence of lower tract infections including the need for hospital admission. Severe disease activity was classified as an oxygen saturation below 94% due to lower tract infection or a respiratory rate above 30 per minute or the need for non-invasive ventilation. Critical disease activity was classified as respiratory failure with the need for invasive ventilation or shock or multiorgan failure or dysfunction. The classification of the various degrees of severity of breakthrough infections is summarized in detail in the Supplemental Table S1.

The Ethics Committee of Heidelberg University approved the study, and the study was registered with the German Clinical Trials Register (DRKS00024632). A written declaration of consent was obtained at the beginning of the study before the application of a booster vaccination.

### 2.2. Assessment of Long-Term Vaccine Responses Using Different Non-Neutralizing and Neutralizing Antibody Assays

To characterize immune responses following adapted vaccination, we conducted various tests to detect antibodies against SARS-CoV-2 epitopes, including assessing their neutralizing capacity. Antibodies against the spike protein and the nucleocapsid protein were detected using commercially available assays (SARS-CoV-2 Total (Siemens) and Elecsys Assay (Roche)). Both assays were performed according to the instructions for use.

To determine the neutralizing ability of antibodies in hemodialysis patients, we performed two different assays. First, we performed an analysis to detect “surrogate” neutralizing antibodies (Medac). The assay is assessing the antibody-mediated inhibition of the interaction between plate-based antigens and the receptor-binding domain of the SARS-CoV-2 virus. The operational mechanism of the assay was recently described in detail by us and others [15,21,22]. In brief, the assay was performed by incubating diluted serum (1 to 10) with RBD conjugated to horseradish peroxidase (HRP) at 37 °C for 20 min, followed by transfer of the mixture to ACE2-coated wells and incubation at 37 °C for 10 min. The interaction between RBD and ACE2 was detected by HRP-induced colorimetric reactions. A signal threshold of ≥30% was considered positive, while a threshold of <30% was considered negative.

Second, we measured the neutralizing ability of serum antibodies against different variants (wildtype and BA.5) in vitro using a model of SARS-CoV-2 infected VeroE6 cells. A detailed description of this assay including the virus isolation, replication and amplification as well as the immunostaining with the determination of neutralizing antibody levels has been provided in detail by Tönshoff et al. [23]. In brief, the wildtype and BA.5 strains of SARS-CoV-2 were cultured and recovered from various swabs from individuals who tested positive by PCR. Vaccine sera were subjected to a twofold dilution and then exposed to either the wildtype strain or the Omicron subvariant. After a one-hour incubation at 37 °C, the mixture was plated onto VeroE6 cells. After 24 h of infection, the cells were fixed and viral replication was assessed using an in-cell ELISA that includes immunostaining for the viral proteins. The results were normalized against the values of cells infected without patient serum and uninfected cells. The neutralizing ability of this assay is expressed as the “inhibitory dilution 50” (ID_50_), which is defined as the dilution of the respective patient serum that results in a 50% reduction of infected cells.

### 2.3. Statistics

Data processing and all analyses were performed using GraphPad Prism software, version 10.2.1. Different antibody levels in the same patients over time were evaluated using the Wilcoxon rank test for matched pairs. Patient characteristics were evaluated using either the Mann–Whitney U test or the χ^2^ test where appropriate. Statistical significance was assumed at *p* < 0.05.

## 3. Results

### 3.1. Long-Term Humoral Immune Response after an Adapted Booster Vaccine Dose in Patients on Hemodialysis

**Figure 2:**
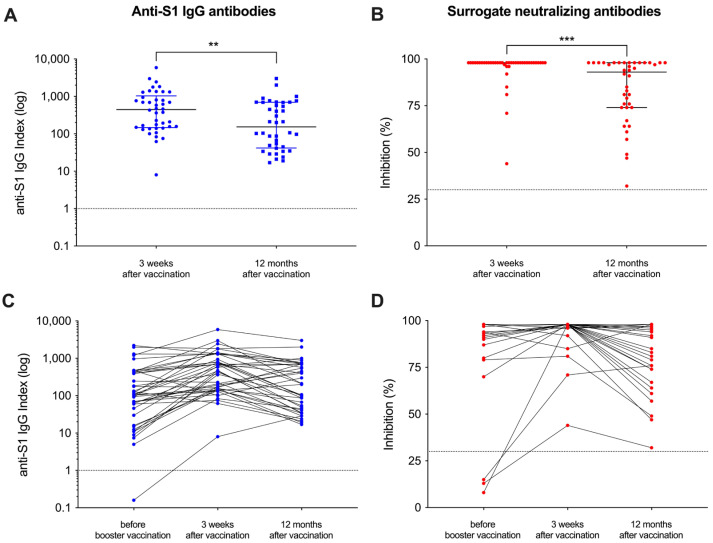
Follow-up of vaccine-induced antibody levels after adapted booster vaccination in patients on hemodialysis over 12 months. Antibody levels against the spike-S1 protein (**A**) and “surrogate” neutralizing antibodies (**B**) were measured 12 months after vaccination and compared to levels three weeks after vaccination. The cutoffs of the assays used are shown by a black line, respectively. Antibody levels around the administration of the adapted vaccine dose and during the 12-month follow-up are shown individually (**C**,**D**). Short-term antibody courses immediately before and 3 weeks after vaccination have been published previously [15]. ** *p* < 0.01; *** *p* < 0.001.

Of the initial 42 patients that had been included in our recent study, 40 patients were eligible for participation in this follow-up study and serum was obtained after a median (IQR) of 370 (361–385) days after the adapted vaccine dose (Figure 1). A detailed analysis of humoral responses and their durability 12 months after booster vaccination was performed. In addition, breakthrough infections during follow-up were assessed in patients with positive anti-nucleocapsid antibodies using clinical data. The clinical characteristics of all hemodialysis patients have recently been published in detail and are shown for the 40 included patients in Supplemental Table S2. With a median (IQR) index of 154 (42–696) and a median (IQR) percent inhibition of 93 (74–98) 12 months after adapted booster vaccination, the anti-S1 IgG antibody levels as well as the surrogate neutralizing antibodies decreased significantly as compared to levels three weeks after vaccination (*p* < 0.01 and *p* < 0.001; Figure 2A,B). However, all patients showed antibody levels exceeding the predefined thresholds for positivity (≥1) and for neutralization (≥30%) after a time course of 12 months post-vaccination. Different antibody levels over time are graphically demonstrated in Figure 2C,D. Before additional booster vaccination, one and three patients on hemodialysis showed no anti-S1 IgG or surrogate neutralizing antibodies, respectively (Figure 2C,D). In contrast, after both 3 weeks and 12 months after vaccination all patients showed levels for both assays above the cutoff, respectively.

### 3.2. In Vitro Neutralization of SARS-CoV-2 Variants 12 Months after Adapted Booster Vaccination

**Figure 3:**
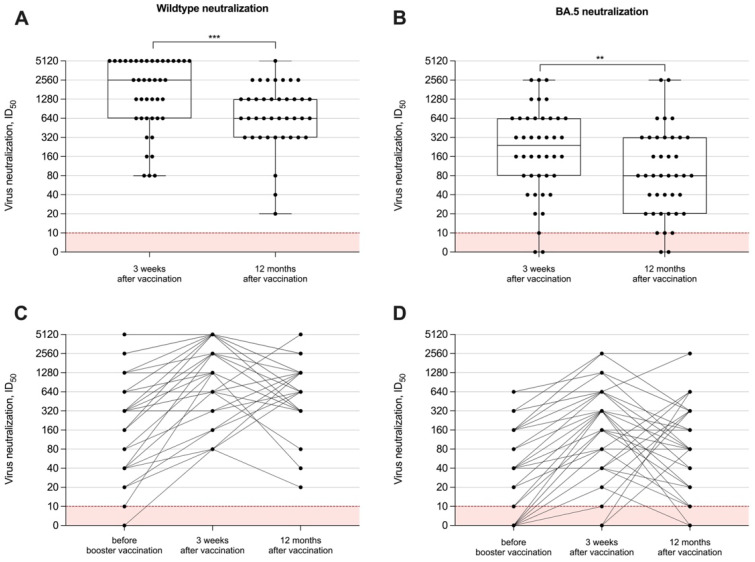
In vitro neutralizing capacity against different SARS-CoV-2 variants over time. The capacity of vaccine-induced antibodies to neutralize the wildtype (**A**) or BA.5 (**B**) has been measured 12 months after booster vaccination with a bivalent vaccine and was compared to levels shortly (3 weeks) after vaccination. In vitro neutralizing capacity around the administration of the adapted vaccine dose and during the 12-month follow-up are shown individually (**C**,**D**). Serum dilution values (ID_50_) of <1:10 are below the detection limit and are shown in red. The short-term neutralizing capacity of antibodies immediately before and 3 weeks after vaccination has been published previously [15]. ** *p* < 0.01; *** *p* < 0.001.

To measure the neutralizing capacity of vaccine-induced antibodies in hemodialysis patients, we performed in vitro tests with different SARS-CoV-2 viral strains, namely the SARS-CoV-2 wildtype and the BA.5 subtype. The long-term neutralization capacity was examined in all 40 individuals after 12 months. Data on humoral immune responses 3 weeks after the adapted booster vaccine dose have been published recently and showed a significant short-term increase for both anti-S1 IgG and surrogate neutralizing antibodies. The ID_50_ for both the wildtype and the BA.5 variant significantly decreased after 12 months with a median (IQR) of 640 (320–1280) and 80 (20–320), as compared to levels three weeks after vaccination with a median (IQR) of 2560 (640–5120) and 240 (80–640), respectively (*p* < 0.001 and *p* < 0.01; Figure 3A,B). All hemodialysis patients had persistent wildtype neutralization activity compared to only 35/40 (88%) with activity against BA.5 12 months after booster vaccination. Individual neutralizing antibody courses over time against both viral strains are shown in Figure 3C,D.

### 3.3. Breakthrough Infections in Hemodialysis Patients after Adapted Booster Vaccination

The COVID-19 disease severity was assessed during the 12-month follow-up using an established grading system, as described in detail in the Supplemental Table S1. Breakthrough infections occurred in 13/40 (33%) individuals, whereas 27/40 (66%) had no breakthrough infection during the 12-month follow-up period. Baseline characteristics of patients with and without breakthrough infections were comparable and are shown in detail in Supplemental Table S3. Notably, 12/13 (92%) hemodialysis patients with breakthrough infections only had asymptomatic or mild disease activity, defined as no or only mild symptoms such as fever, cough, change in sense of smell and taste, no occurrence of dyspnea and no need for hospitalization. One patient had moderate disease activity, defined as clinical or radiographic evidence of lower tract infections including the need for hospital admission but no oxygen saturation below 94%, no respiratory rate above 30 per minute and no need for non-invasive ventilation. The patient required hospitalization but could be discharged from hospital after 5 days without requiring intensive care support.

**Figure 4:**
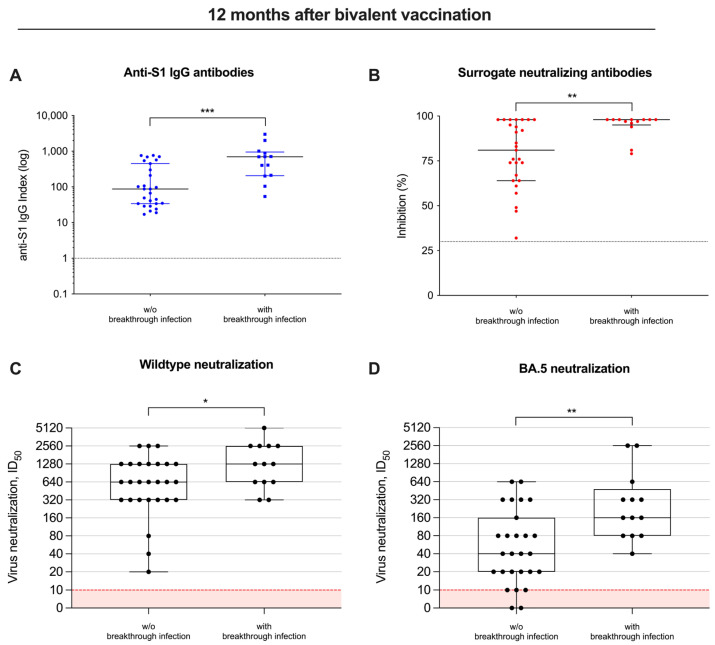
Different vaccine-induced antibodies, including their neutralizing activity in patients with and without breakthrough infections. Antibody levels against the spike S1 protein (**A**), antibodies with “surrogate” neutralizing capacity (**B**) and in vitro neutralization of virus strains (**C**,**D**) were measured in patients with and without breakthrough infections during follow-up 12 months after bivalent vaccination. The detection limits are illustrated by red lines for each assay, respectively. * *p* < 0.05; ** *p* < 0.01; *** *p* < 0.001.

Twelve months after bivalent vaccination, anti-S1 antibodies were with a median (IQR) index of 697 (206–950) as compared to 87 (34–450), significantly higher in individuals with breakthrough infections as compared to individuals without breakthrough infections during follow-up (*p* < 0.001; Figure 4A). Similarly, surrogate neutralizing antibodies were also significantly higher with a median (IQR) percent inhibition of 98 (95–98) in patients with breakthrough infections as compared to a median (IQR) of 81 (64–98) in patients without breakthrough infections (*p* < 0.01; Figure 4B). 

Consequently, the ID_50_ for both the wildtype and BA.5 significantly increased 12 months after bivalent vaccination, and was—with a median (IQR) of 1280 (640–2560) and 160 (80–480)—significantly higher in patients with breakthrough infections, compared to a median (IQR) of 640 (320–1280) and 40 (20–160) in individuals without breakthrough infections (*p* < 0.05 and *p* < 0.01; Figure 4C,D).

## 4. Discussion

Data on the durability of protective antibodies following an adapted vaccine dose in hemodialysis patients are urgently needed to help guide vaccination booster intervals. In addition, studies on the frequency and severity of breakthrough infections within this vulnerable cohort are still scarce. This study is an extension of previously published short-term data on humoral immune responses following an adapted booster vaccine dose in patients on hemodialysis. We conducted a long-term follow-up of humoral immune responses 12 months after vaccination, and conclusively demonstrated that the use of an adapted vaccine induced a sustained immune response with protective neutralizing antibodies in most patients over the follow-up period.

As expected, with commercially available tests, we found a significant decrease in antibodies against the spike protein and surrogate neutralizing antibodies 12 months after bivalent vaccination. Yet, although live-virus neutralizing antibodies against different variants also decreased significantly over time, the proportion of hemodialysis patients with detectable antibody levels after 12 months was 100% and 85% for the wildtype and BA.5, respectively. Despite the robust response to vaccination, breakthrough infections occurred in 13 out of 40 hemodialysis patients during the 12-month follow-up, most likely due to the high infectivity of different SARS-CoV-2 Omicron subtypes [24]. However, most breakthrough infections were asymptomatic or mild and only one was classified as moderate disease activity, attesting good vaccine efficacy, as shown previously already for healthy cohorts [25]. Individuals who experienced breakthrough infections showed significantly higher levels of neutralizing antibodies against different SARS-CoV-2 subtypes compared to those without breakthrough infections during follow-up.

Booster mRNA vaccine doses have been shown to significantly increase both humoral and cellular immunity in the general population as well as in immunocompromised individuals such as patients on hemodialysis [26,27,28]. It has already been shown that a pronounced, albeit lower, vaccination response could be achieved after a standard double immunization with an mRNA vaccine in patients on hemodialysis compared to the healthy population [2]. These vaccination responses could then be successively increased through various vaccination programs such as further non-adapted booster vaccinations or the combination of mRNA- and vector-based vaccines [29,30].

Emerging Omicron subtypes with at least partial immune escape have led to the rapid advancement of research into bivalent vaccines. Studies by us and others showed high antibody levels against different spike epitopes of emerging variants of concern after adapted booster vaccination in immunocompromised patients on hemodialysis [15,31]. In our initial study, on which this recent extension is based, we showed that hemodialysis patients without prior SARS-CoV-2 infection from before to 3 weeks after a fifth dose with the adapted booster vaccine had significantly improved antibody neutralization against wildtype and BA.5 [15]. In addition, data published by Bronder et al. revealed a significant induction of spike-specific T-cells after bivalent vaccination, with even higher levels in hemodialysis patients after SARS-CoV-2 infection [30]. These data are consistent with data from healthy individuals in whom a strong and versatile cellular immune response is triggered after administration of COVID-19 vaccines, especially after mRNA-based vaccinations [31]. These vaccines have been shown to activate both CD4^+^ and CD8^+^ T lymphocytes, which play a central role in cell-mediated immunity. This dual activation also facilitates the synthesis of high affinity neutralizing antibodies, indicating the important interplay between humoral and cellular immunity following vaccination. In addition, the breadth and heterogeneity of the T cell response, which includes multiple viral target epitopes, increases the overall efficacy of the immune defense and reduces the potential viral immune escape [32,33].

Manley et al. investigated the protective effect of vaccination against severe COVID-19 disease, including data on hospitalization and mortality in immunocompromised hemodialysis patients. Both breakthrough infections and disease severity including fatal outcomes were significantly reduced after vaccination and inversely correlated with antibody levels [3]. Karoui et al. also showed that vaccination had a protective effect on COVID-19 disease severity despite the reported lower humoral and cellular immune responses in dialysis patients as compared to healthy individuals [34]. Our results support those by Manley et al. and Karoui et al., as antibody levels and their neutralizing capacity were high in most individuals after bivalent vaccination and no severe breakthrough infections were detected during follow-up. However, the incidence of breakthrough infections was, at 33%, relatively high during the 12-month follow-up period, indicating that the transmission of different Omicron variants was not entirely averted, despite partly high antibody levels. Notably, the only individual with a moderate breakthrough infection requiring hospitalization had no detectable in vitro neutralization against BA.5 directly after the booster vaccination. These findings again highlight the importance of COVID-19 vaccination and provide a rationale for further studies investigating vaccine responses in this immunocompromised cohort to help guide further vaccination recommendations. 

One major limitation of our study is the lack of data on cellular immunity. Another limitation is the small sample size, which particularly limits statements about the significance of breakthrough infections and their severity. Our study was designed to characterize humoral immune responses, including data on in vitro neutralization. Larger follow-up studies with breakthrough infections as the primary endpoint are needed to provide robust recommendations for further booster vaccinations in this high-risk cohort. Another general limitation of studies investigating SARS-CoV-2 vaccination is a still unknown correlate of protection against breakthrough infections or severe disease courses, necessitating further validation.

In summary, the bivalent booster vaccination appears to induce a sustained immune response with robust neutralizing antibodies in the majority of hemodialysis patients over a period of 12 months. During the follow-up period, several, but mostly asymptomatic or mild breakthrough infections occurred. Because neutralizing antibody levels have been shown to be highly predictive of protection against severe COVID-19 disease courses, this study adds further evidence for recommending bivalent vaccination in this high-risk population to achieve long-term protection against severe breakthrough infections. However, further epidemiological studies addressing the incidence and severity of breakthrough infections in larger cohorts are needed to support further booster vaccinations in hemodialysis patients.

## Data Availability

The raw data supporting the conclusions of this article will be made available by the authors on request.

## Supplementary Materials

The following supporting information can be downloaded at: https://www.mdpi.com/article/10.3390/vaccines12070738/s1, Supplemental Table S1. Definition of COVID-19 disease severity; Supplemental Table S2. Patients’ characteristics; Supplemental Table S3. Demographic and clinical characteristics of hemodialysis patients with and without SARS-CoV-2 infection after an adapted SARS-CoV-2 vaccine dose.
