# Supplementary material for: Durability of Humoral Responses after an Adapted SARS-CoV-2 mRNA Vaccine Dose in Hemodialysis Patients
Source: Vaccines (Basel). 2024 Jul 3;12(7):738. doi: 10.3390/vaccines12070738 (PMC11281374; doi:10.3390/vaccines12070738)
Supplement: Supplementary file 1 [file vaccines-12-00738-s001.zip › vaccines-3004527-supplementary.pdf]

Supplemental data

Supplemental Table S1. Definition of COVID-19 disease severity

COVID-19 Severity	Endpoint definitions
Asymptomatic/Mild	Asymptomatic or only mild symptoms (i.e. fever, cough, change in taste of smell), no dyspnea; no need for hospital admission
Moderate	Clinical or radiographic evidence of lower respiratory tract disease; need for hospital admission
Severe	Oxygen saturation <94%, respiratory rate ≥30 breaths/min; need for non-invasive ventilation
Critical	Respiratory failure, shock, multiorgan dysfunction or failure; need for invasive ventilation

Supplemental Table S2. Patients' characteristics.

	Hemodialysis patients, (N=40)
Age (years), median (IQR)	73 (62–81)
Sex	
Female, N (%)	11 (28)
Male, N (%)	29 (73)
Dialysis vintage (years), median (IQR)	5 (3–8)
Cause of end-stage kidney disease	
Diabetes, N (%)	8 (20)
Vascular, N (%)	14 (35)
Polycystic kidney disease, N (%)	3 (8)
Glomerulonephritis, N (%)	9 (23)
Chronic pyelonephritis, N (%)	1 (3)
Other, N (%)	5 (13)
Comorbidities	
Diabetes mellitus, N (%)	14 (35)
Hypertension, N (%)	36 (90)
Obesity (BMI >25 kg/m^2), N (%)	27 (68)
Coronary heart disease, N (%)	19 (48)
Peripheral artery disease, N (%)	11 (28)
Chronic heart failure, N (%)	11 (28)
COPD, N (%)	6 (15)
Liver Cirrhosis, N (%)	3 (8)

BMI, body mass index; COPD, chronic obstructive pulmonary disease; IQR, interquartile range.

Supplemental Table S3. Demographic and clinical characteristics of hemodialysis patients with and without SARS-CoV-2 infection after an adapted SARS-CoV-2 vaccine dose.

	With breakthrough infection, N=27	Without breakthrough infection, N=13	*P*-value
Age (years), median (IQR)	79 (68–82)	74 (62–80)	0.51
Sex			
Male, n (%)	18 (67)	10 (77)	0.78
Female, n (%)	9 (33)	3 (23)	
Dialysis vintage (years), median (IQR)	4 (2–6)	6 (3–9)	0.43
Cause of end-stage kidney disease			
Diabetes, N (%)	4 (15)	4 (31)	0.32
Vascular, N (%)	8 (30)	6 (46)	0.56
Polycystic kidney disease, N (%)	3 (11)	0 (0)	N/A
Glomerulonephritis, N (%)	5 (19)	4 (31)	0.48
Chronic pyelonephritis, N (%)	0 (0)	1 (8)	N/A
Other, N (%)	2 (7)	3 (23)	0.19
Comorbidities			
Diabetes mellitus, n (%)	8 (30)	6 (46)	0.45
Hypertension, n (%)	24 (89)	12 (92)	0.88
Obesity (BMI >25 kg/m^2), n (%)	19 (70)	8 (62)	0.64
Coronary heart disease, n (%)	14 (52)	5 (38)	0.57
Peripheral artery disease, n (%)	9 (33)	2 (15)	0.33
Chronic heart failure, n (%)	7 (26)	4 (31)	0.86
COPD, n (%)	3 (11)	3 (23)	0.49
Cirrhosis, n (%)	2 (7)	1 (8)	0.90

BMI, body mass index; COPD, chronic obstructive pulmonary disease; IQR, interquartile range; N/A, not aplicable
